# Factors associated with hand joint destruction in Chinese patients with rheumatoid arthritis

**DOI:** 10.1186/s12891-017-1548-7

**Published:** 2017-05-22

**Authors:** Lijuan Zhang, Jing wang, Qiuxiang Zhang, Ting Fu, Rulan Yin, Ze Wang, Liren Li, Xianhua Wu, Zhifeng Gu

**Affiliations:** 1 Department of Rheumatology, Affiliated Hospital of Nantong University, 20th Xisi Road, 226001 Nantong, People’s Republic of China; 2 School of Nursing, Nantong University, 19th Qixiu Road, 226001 Nantong, People’s Republic of China; 3 Department of Medical Image, Affiliated Hospital of Nantong University, 20th Xisi Road, 226001 Nantong, People’s Republic of China

**Keywords:** Rheumatoid arthritis, Hand joint destruction, Disease activity, Quality of life

## Abstract

**Background:**

There have been no previous report of hand joint destruction prevalence in Chinese rheumatoid arthritis (RA) patients. Therefore, the aim of this study was to investigate the prevalence and potential factors of hand joint destruction among RA patients from Nantong China. In addition, we wanted to examine the differences between functional capacity, psychological status, and quality of life in patients with hand joint destruction compared to those without hand joint destruction.

**Methods:**

A cross-sectional study was conducted from the Affiliated Hospital of Nantong University between July 2015 and June 2016. RA patients completed questionnaires for demographic or clinical variables, the 10-cm Visual Analog Scale for pain, the 28-joint Disease Activity Score-erythrocyte sedimentation rate for disease activity, the Health Assessment Questionnaire-disability index for physical function, the Hospital Anxiety and Depression Scale for anxiety and depression, and the Short Form 36 health survey for quality of life. Laboratory examinations were taken to obtain some biochemical indicators (e.g., rheumatoid factor, anti-cyclic citrullinated peptide antibody). X-ray assessment of hand was performed and hand joint destruction was defined as Sharp score > 0. Independent sample *t*-test, Mann–Whitney *U*-test, Chi-square test, and multivariate analysis using backward stepwise logistic regression model were used to analyze these data.

**Results:**

One hundred and sixty-one RA patients were included in this study. Radiographic findings revealed that almost 47.2% (*n* = 76) of patients had hand joint destruction. Multivariate analysis found that education ≤ 9 years (*p* = 0.041), anti-cyclic citrullinated peptide antibody positive (*p* = 0.021), high disease activity (*p* = 0.020), and long disease duration (*p* < 0.001) were important potential risk factors of hand joint destruction. Participants with hand joint destruction tended to have lower physical function and quality of life, and more severe depressive symptoms compared to individuals without hand joint destruction.

**Conclusions:**

47.2% of people with RA from Nantong China experienced hand joint destruction. Education, anti-cyclic citrullinated peptide antibody, disease activity, and disease duration had great impacts on hand joint destruction. The results suggested that rheumatologists should pay attention to RA patients’ hand joint destruction, especially those with low education levels, anti-cyclic citrullinated peptide antibody positive, high disease activity, and long disease duration by patient education or other ways to improve patients’ prognosis.

## Background

As one of the most common clinical manifestations among rheumatoid arthritis (RA) patients, joint destruction is a serious threat to physical function [1, 2] and quality of life [3] for this population. Recent investigations have reported that people with RA have an increased risk of experiencing joint destruction with the prolongation of the course of disease [4]. This highlight the fact that joint destruction must be understood in order to inhibit radiographic progression in RA patients. Current epidemiological evidence suggested that female [5], old age [6], low body mass index (BMI) [7, 8], low socioeconomic status (SES) [9], less alcohol usage [10, 11], long disease duration [7, 12], high disease activity [13, 14], and comorbidities [15, 16] were associated with joint destruction. For many years, rheumatoid factor (RF) [17–20] and anti-citrullinated peptide (CCP) antibody [21–23] have been regarded as important risk factors for joint destruction in RA patients. Additionally, drug treatments play important roles in preventing RA patients from joint destruction [24–26]. Therefore, we were specifically interested in the association between those variables and hand joint destruction. However, there have been no previous report of hand joint destruction prevalence in Chinese RA patients. Thus, the aim of this study was to explore the prevalence and potential risk factors of hand joint destruction among RA patients from Nantong China. In addition, we wanted to examine the differences between functional capacity, psychological status, and quality of life in patients with hand joint destruction compared to those without hand joint destruction.

## Methods

### Patients

All patients fulfilled the 1987 revised criteria of the American College of Rheumatology (ACR) [27] or 2010/2012 ACR classification criteria [28, 29] for RA. They were recruited from the Affiliated Hospital of Nantong University between July 2015 and June 2016. RA patients enrolled during study period were all patients who visited their rheumatology clinic (outpatient or inpatient department). Patients included in this study were treated in accordance with the 2008 ACR recommendations for the management of RA [30]. Patients meeting the following exclusion criteria were excluded: (1) they aged less than 18 years old; (2) they could not complete the questionnaires; (3) they had specific comorbidities including renal, serious cardiac, liver diseases or malignancy that could influence their quality of life; (4) they could not complete the measurements of hand joint destruction, disease activity or pain. This cross-sectional study was approved by the Affiliated Hospital of Nantong University, and a written informed consent was obtained from each RA patient according to the Declaration of Helsinki.

### Demographic and clinical characteristics

Demographic and clinical data included gender, age (years), BMI (kg/m^2^), education (years), employment status, income/person/month (USD), personal health insurance, tobacco and alcohol usage, comorbidities (e.g., hypertension), and disease duration (years). Several serological markers including C-reactive protein (CRP), erythrocyte sedimentation rate (ESR), RF and anti-CCP antibody were examined in this study. We evaluated disease activity using the 28-joint Disease Activity Score-ESR (DAS28-ESR) [31]. All demographic, clinical, laboratory data and radiographs were collected at the same time point. We gained the personal medication by querying the electronic medical records and patients’ self-reports, including the usages of disease-modifying antirheumatic drugs (DMARDs), corticosteroids and biologics from the past six months to the date of radiographs.

### Assessment of patient-reported outcomes

Patients’ pain was assessed by the 10-cm visual analog scale (VAS) (0 = no pain, and 10 = most severe pain) [32]; The Health Assessment Questionnaire-disability index (HAQ-DI) was used to assess physical function [33]; As described previously [34], the Hospital Anxiety and Depression Scale (HADS), a 14-item questionnaire, was used to assess levels of anxiety and depression. Anxiety and depression are scored separately using the 7-item subscales (scores range from 0–21, with higher scores indicating more severe mood disorders). As described previously [35], quality of life was assessed using the Short Form 36 health survey (SF-36), which assesses eight domains (scores range from 0–100, with higher scores indicating better health status): physical functioning (PF), role physical (RP), bodily pain (BP), general health (GH), vitality (VT), social functioning (SF), role emotional (RE), and mental health (MH). Z-transformed and normalized domain scores are grouped into Physical Component Summary (PCS) and Mental Component Summary (MCS) scores.

### Radiographic assessments

All radiographs were scored in accordance with the van der Heijde-modified Sharp Score of hands by two experienced investigators (GZ and WXH) who were not aware of the patients’ clinical findings [36, 37]. The total score for the hands, including the wrists, 10 metacarpophalangeal joints (MCPJs), and 10 proximal interphalangeal joints (PIPJs), ranged from 0 to 157, with the erosion score (E score) ranging from 0 to 85, and the joint space narrowing score (JSN score) ranging from 0 to 72. Hand joint destruction was categorized as following: normal (Sharp score = 0), abnormal (Sharp score > 0).

### Data collection

Questionnaires and some measurements were administered to patients from July 2015 to June 2016. The written questionnaire was completed by the patients with the physician present or the questionnaire was completed by the physician asking the patients questions (an interview-led questionnaire) in a clinical setting. The same clinician evaluated DAS28-ESR for all patients. After finishing data collection, nurses would calculate the results. Two research assistants added the results to a computer database by double checked against the original data.

### Statistical analysis

Because there were two patients lacking some socio-demographic data in this study, we used multiple imputation (MI) to handle missing data. As described previously [38], MI is a general approach to the problem of missing data that is available in several commonly used statistical packages. The first stage is to create multiple copies of the dataset, with the missing values replaced by imputed values. The second stage is to use standard statistical methods to fit the model of interest to each of the imputed datasets. In this study, i) five imputed datasets were created, ii) the multiple imputation procedure in SAS statistical software (PROC MI) was used to impute the missing data and iii) employment status, education and tobacco usage were included in the imputation models.

We conducted descriptive analyses to investigate the patients’ characteristics. Continuous and normally distributed variables were presented as means and standard deviation (SD) and independent sample *t*-test was used to assess group differences. Not normally distributed data was described by median and interquartile range (IQR) and Mann–Whitney *U*-test was used to assess group differences. Descriptive statistics also involved frequencies (%) for categorical variables and the chi-square test was used to assess group differences. Variables shown to be significant in the independent sample *t*-test, Mann–Whitney *U*-test or chi-square test were included in the multivariate analysis using backward stepwise logistic regression model to investigate the potential risk factors of hand joint destruction with odds ratios (ORs), and the corresponding 95% confidence intervals (CIs). The backward stepwise logistic regression model fit was evaluated by the Hosmer-Lemeshow goodness-of-fit test. Statistical significance was considered when *p* < 0.05 (two-sided). Data were analyzed using SPSS (version 20.0).

## Results

### Patient characteristics

**Fig. 1:**
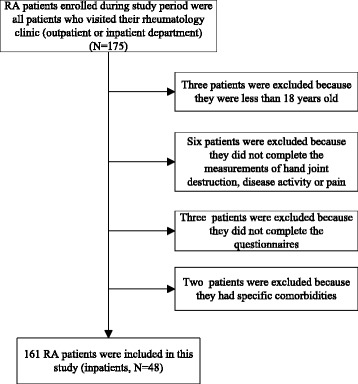
“Flow chart of the study”

Fourteen RA patients met the exclusion criteria, resulting in the enrollment of 161 RA patients in the current study, of which 48 patients were inpatients (Fig. 1). Table 1 presented the baseline participant characteristics included in our analysis. The mean (SD) age of the respondents was 53.7 (12.9) years, and 83.9% were female. The median (IQR) of disease duration and DAS28-ESR were 4.1 (9) years and 3.3 (1.7), respectively. Almost 94.4% of patients were treated with DMARDs. There were 67.7% of patients who were RF positive and 64.6% of patients who were anti-CCP antibody positive. Radiographic finding revealed that almost 47.2% (*n* = 76) of individuals in the sample group had hand joint destruction. The range of Sharp scores in the patients with hand joint destruction was from 1 to 117.

**Table 1:** Differences between demographic and clinical variables in patients with hand joint destruction compared to those without hand joint destruction

Variables	Overall sample (*N* = 161)	Non-hand joint destruction (Sharp score = 0; *N* = 85)	Hand joint destruction (Sharp score > 0; *N* = 76)	*χ* ^2^/t/z	*P*-value
Demographic factors					
Female gender, no. (%)	135 (83.9)	69 (81.2)	66 (86.8)	0.95^*^	0.329
Age, mean (SD) years	53.7 (12.9)	52.7 (13.7)	54.8 (12.1)	−1.03^**^	0.303
BMI, mean (SD) kg/m^2^	22.0 (3.4)	22.3 (3.3)	21.6 (3.5)	−1.29^**^	0.198
Education, years, no. (%)				5.58^*^	0.018
≤ 9 years	122 (75.8)	58 (68.2)	64 (84.2)		
> 9 years	39 (24.2)	27 (31.8)	12 (15.8)		
Employment status, no. (%)				0.01^*^	0.946
Employed	97 (60.2)	51 (60)	46 (60.5)		
Unemployed	64 (39.8)	34 (40)	30 (39.5)		
Income/person/month, USD, no. (%)				0.28^*^	0.599
≤ 435 USD	144 (89.4)	75 (88.2)	69 (90.8)		
> 435 USD	17 (10.6)	10 (11.8)	7 (9.2)		
Personal health insurance, no. (%)				0.07^*^	0.785
Yes	117 (72.7)	61 (71.8)	56 (73.7)		
No	44 (27.3)	24 (28.2)	20 (26.3)		
Tobacco usage, no. (%)				2.42^*^	0.120
Yes	17 (10.6)	12 (14.1)	5 (6.6)		
No	144 (89.4)	73 (85.9)	71 (93.4)		
Alcohol usage, no. (%)				1.66^*^	0.197
Yes	23 (14.3)	15 (17.6)	8 (10.6)		
No	138 (85.7)	70 (82.4)	68 (89.4)		
Clinical factors					
Comorbidities, yes, no. (%)				2.34^*^	0.127
Yes	34 (21.1)	14 (16.5)	20 (26.3)		
No	127 (78.9)	71 (83.5)	56 (73.7)		
Disease duration, median (IQR) years	4.1 (9)	2 (4.5)	9.5 (13.5)	−6.64	<0.001
VAS pain (range 0–10), median (IQR)	5 (6)	3 (6)	5 (6.5)	−1.05	0.294
DAS28-ESR, median (IQR)	3.3 (1.7)	2.9 (1.6)	3.6 (1.8)	−3.35	0.001
DMARDs usage, no. (%)				4.98^*^	0.026
Yes	152 (94.4)	77 (90.6)	75 (98.7)		
No	9 (5.6)	8 (9.4)	1 (1.3)		
Corticosteroids usage, no. (%)				9.32^*^	0.002
Yes	92 (57.1)	39 (45.9)	53 (69.7)		
No	69 (42.9)	46 (54.1)	23 (30.3)		
Biologics usage, no. (%)				0.02^*^	0.889
Yes	11 (6.8)	3 (3.5)	3 (3.9)		
No	150 (93.2)	82 (96.5)	73 (96.1)		
ESR, median (IQR) mm/h	22 (38)	20 (35.5)	23.5 (50.5)	−0.90	0.367
CRP, median (IQR) mg/l	7.5 (12.4)	5.9 (13.6)	8.2 (20.6)	−1.60	0.110
RF positive, no. (%)				12.68^*^	<0.001
Yes	109 (67.7)	47 (55.3)	62 (81.9)		
No	52 (32.3)	38 (44.7)	14 (18.1)		
Anti-CCP antibody positive, no. (%)				15.45^*^	<0.001
Yes	104 (64.6)	43 (50.6)	61 (80.3)		
No	57 (35.4)	42 (49.4)	15 (19.7)		

*IQR* Interquartile range, *BMI* body mass index, *VAS* visual analog scale, *DAS28* Disease Activity Score in 28 joints, *DMARDs* disease-modifying antirheumatic drugs, *ESR* erythrocyte sedimentation rate, *CRP* C-reactive protein, *RF* rheumatoid factor, *Anti-CCP* anti-cyclic citrullinated peptide

^*^Chi-square test

^**^Independent t-tests

### Differences between demographic and clinical variables in patients with hand joint destruction compared to those without hand joint destruction

As shown in Table 1, a number of demographic and clinical variables were tested for possible differences in patients with hand joint destruction compared to those without hand joint destruction. The patients were divided into two groups according to Sharp score. Patients with hand joint destruction tended to have lower education level (≤9 years) (*p* = 0.018), longer disease duration (*p* < 0.001), higher disease activity (*p* = 0.001), DMARDs usage (*p* = 0.026), corticosteroids usage (*p* = 0.002), RF positive (*p* < 0.001), and anti-CCP antibody positive (*p* < 0.001) compared to patients without hand joint destruction. However, no statistically significant associations were found with regard to comorbidities (*p* = 0.127), VAS pain (*p* = 0.294), biologics usage (*p* = 0.889), ESR *(p* = 0.367), and CRP (*p* = 0.110).

### Multivariate analysis using backward stepwise logistic regression model for hand joint destruction

We used the multivariate analysis using backward stepwise logistic regression model to investigate the potential risk factors of hand joint destruction, as indicated in Table 2. The result of final step indicated that low education level (≤9 years) (OR: 3.06; *p* = 0.041), anti-CCP antibody positive (OR: 1.07; *p* = 0.021), high disease activity (OR: 1.45; *p* = 0.020), and long disease duration (OR: 1.21; *p* < 0.001) were the potential risk factors of hand joint destruction in RA patients from Nantong China. The model (step 2) had a good fit under the Hosmer- Lemeshow goodness-of-fit test (*R* ^*2*^ = 0.379, *χ* ^2^ = 8.26, *p* = 0.411).

**Table 2:** Results of multivariate analysis using backward stepwise logistic regression model in patients with hand joint destruction

Variables		B	SE	Wald	OR	OR 95% CI		*P*-value
						Lower bound	Upper bound	
Step 1	Education ≤ 9 years	1.11	0.54	4.26	3.01	1.16	7.64	0.038
	Disease duration, years	0.18	0.05	20.37	1.22	1.13	1.51	<0.001
	DAS28-ESR	0.39	0.16	5.30	1.46	1.06	2.15	0.022
	DMARDs usage, yes	−2.24	1.28	3.11	0.13	0.04	1.37	0.077
	Corticosteroids usage, yes	−0.76	0.45	3.06	0.48	0.21	1.09	0.080
	RF positive, yes	−0.66	0.39	2.79	0.63	0.13	1.74	0.095
	Anti-CCP antibody positive, yes	−0.97	0.46	5.02	1.03	0.17	1.57	0.025
R^2^		0.380						
Step 2	Education ≤ 9 years	1.11	0.55	4.24	3.06	1.1	8.41	0.041
	Disease duration, years	0.20	0.05	21.26	1.21	1.12	1.32	<0.001
	DAS28-ESR	0.38	0.16	5.43	1.45	1.16	2.17	0.020
	Anti-CCP antibody positive, yes	−0.99	0.47	5.12	1.07	0.16	1.68	0.021
R^2^		0.379						

*OR* odds ratio, *CI* confidence interval, *DAS28-ESR* Disease Activity Score in 28 joints-erythrocyte sedimentation rate, *DMARDs* disease-modifying antirheumatic drugs, *RF* rheumatoid factor, *Anti-CCP* anti-cyclic citrullinated peptide

### Differences between functional capacity, psychological status, and quality of life in patients with hand joint destruction compared to those without hand joint destruction

We found that the HAQ-DI and HADS-depression scores in individuals with hand joint destruction were significantly higher compared to individuals without hand joint destruction. However, expect the PF (*p* = 0.012), no statistically significant association was found with regard to other dimension scores of SF-36 (Table 3).

**Table 3:** Differences between patient-reported outcomes in patients with hand joint destruction compared to those without hand joint destruction

Variables	Overall sample (*N* = 161)	Non-hand joint destruction (Sharp score = 0; *N* = 85)	Hand joint destruction (Sharp score > 0; *N* = 76)	z	*P*-value
HAQ-DI score (range 0–3), median (IQR)	0.15 (0.88)	0.05 (0.55)	0.25 (1.26)	−3.62	<0.001
HADS-anxiety score (range 0–21), median (IQR)	5 (3)	5 (6)	5 (5)	−0.57	0.571
HADS-depression score (range 0–21), median (IQR)	5 (5)	3 (4.5)	6 (6)	−1.50	0.047
Domains of SF-36 scores (range 0–100), median (IQR)					
PCS	37.75 (35.25)	38 (32.75)	36.63 (34.13)	−1.34	0.180
MCS	58.13 (35.13)	59.63 (36.5)	56.96 (34.22)	−0.80	0.423
PF	60 (47.5)	65 (52.5)	55 (55)	−2.50	0.012
RP	0 (25)	0 (25)	0 (25)	−0.22	0.830
BP	41 (43.5)	52 (42)	41 (51.5)	−0.78	0.434
GH	40 (32)	42 (31.5)	40 (30)	−1.44	0.106
VT	55 (20)	55 (22.5)	60 (20)	−0.32	0.752
SF	62.5 (50)	62.5 (50)	62.5 (46.88)	−1.26	0.208
RE	33.3 (100)	66.7 (100)	33.3 (100)	−0.32	0.752
MH	64 (24)	68 (24)	64 (24)	−0.30	0.761

*IQR* Interquartile range, *HAQ-DI* health assessment questionnaire-disability index, *HADS* Hospital Anxiety and Depression Scale, *SF-36* Short Form 36 health survey, *PCS* physical components summary, *MCS* mental components summary, *PF* physical functioning, *RP* role physical, *BP* body pain, *GH* general health, *VT* vitality, *SF* social functioning, *RE* role emotional, *MH* mental health

## Discussion

To our knowledge, this is the first study investigating the prevalence and potential risk factors (e.g., education, anti-CCP antibody) of hand joint destruction in RA patients from Nantong China. As described previously [39], x-ray assessment of hand/wrist was repeated at baseline and the 12th month and a change of total Sharp score > 0.5 units was defined as radiographic progression. Because this study was cross-sectional in design, we defined hand joint destruction according to the van der Heijde-modified Sharp Score and their cutoff value was 0, which was a quite understandable indicator for hand joint destruction. In the current study, almost 47.2% of RA patients from Nantong China suffered from hand joint destruction. This prevalence estimate is significantly lower than those observed both in China [39] and other countries [5]. Such discrepancy could be explained that the majority of RA patients included in this study were outpatients with milder disease, which would have led to a lower prevalence estimate.

Previous studies have reported that female [5], older age [6], and less alcohol usage [10, 11] were associated with joint destruction. However, our study demonstrated that there were no correlation between these variables and hand joint destruction. One possible explanation for the different results is the existence of cultural diversity between Chinese and Western population. It must be noted that there was no association between BMI and hand joint destruction in this study, which was in contrast with previous meta-analysis reporting that obesity (BMI > 30 kg/m^2^) was associated with lower radiographic joint damage in RA [8]. It could be explained that the mean BMI (22 kg/m^2^) in this study was much lower than that in previous study [8]. Furthermore, we reported that comorbidities were not associated with hand joint destruction, which was in contrast with previous studies [15, 16]. This differences might be explained that certain patients with comorbidities were excluded in this study. Additionally, it is well known that SES is multifactor (e.g., education, income) and low SES could result in joint destruction in RA patients [9]. Our study also demonstrated that there were significant correlation between low education level and hand joint destruction. It might be explained that patients with low education levels tended to have low medication adherence rates [40], which could result in hand joint destruction.

Not surprisingly, disease duration was significantly associated with hand joint destruction in RA patients from Nantong China, which was in line with previous studies [7, 12, 19]. When RA progresses, the joints will be more and more affected (e.g., bone erosion), which possibly result in hand joint destruction. We also found that disease activity was significantly associated with hand joint destruction, which was similar to previous studies [13, 14]. Our group has reported that about 57% patients tended to take 2–3 DMARDs in Chinese RA patients [40]. In the current study, we reported that DMARDs usage had a positive correlation with hand joint destruction. It might be explained that those using more than one DMARDs could be patients with more severe disease, which could lead to more severe joint destruction [41–44]. In contrast with previous meta-analysis [45], we found that corticosteroids usage was positively associated with hand joint destruction, which might be explained that RA patients using corticosteroids might be those with difficult to control inflammation, or who could not take DMARDs. In addition, we reported that only 6.8% of RA patients from Nantong China used biologics. The proportion of biologics usage is much lower than that in previous studies from other countries [46, 47]. It could be explained that Chinese RA patients with low SES cannot afford the costs of biologics, which are expensive and cannot be reimbursed by person health insurance. In accordance with previous studies [18, 19, 21–23], our study demonstrated significant correlations among RF, anti-CCP antibody, and hand joint destruction, which could be explained that RF and anti-CCP antibody positive were both important predictors of structural progression in RA patients. However, some analysis reported a higher correlation of anti-CCP antibody with joint damage than of RF [21, 23]. Therefore, to identify which variables were most significantly correlated with hand joint destruction, the multivariate analysis using backward stepwise logistic regression model was used. We found that education ≤ 9 years, anti-CCP antibody positive, high disease activity, and longer disease duration were the potential risk factors for hand joint destruction.

There are, however, additional important shortcomings in this study that need to be addressed. First, all patients involved in this investigation were only from one center and the sample size was relatively small, so generalization of the findings to other population should be cautious. In addition, the majority of patients were female and some of them were inpatients, therefore, our sample was not representative of the Chinese RA population. Another problem with the sampling method was that some patients with milder disease might be missed (less likely to be an inpatient, less likely to be attending outpatient reviews very often, so therefore possibly less likely to be invited to participate in this study). Second, the inter-rater reliability of Sharp score also could not be tested. However, to ensure the accuracy of Sharp score for the hands, two experienced observers evaluated hand joint destruction according to the HSS at the same time, and all readers were blind to the results. Third, all patients were heterogeneous in disease duration, which could result in selection bias. Therefore, more detailed analysis was needed to address the potential risk factors such as early diagnosis and immediate treatment for early radiographic changes of hands in RA patients. Fourth, slightly different methods were used to complete the questionnaires (patients completed with physician present versus physician-led completion) in this study, which might have introduced measurement bias. However, the survey interviewers were professionally trained in order to reduce this bias. Finally, causal conclusion could not be inferred because this study was cross-sectional in design.

## Conclusions

47.2% of people with RA from Nantong China experienced hand joint destruction. Education, anti-CCP antibody, disease activity, and disease duration played important roles in the prevalence of hand joint destruction. The results suggested that rheumatologists should pay attention to RA patients’ hand joint destruction, especially those with low education levels, anti-CCP antibody positive, high disease activity, and long disease duration by patient education or other ways to improve patients’ prognosis.

## Abbreviations

ACR: American College of Rheumatology
anti-CCP: Anti-Cyclic Citrullinated Peptide
BMI: Body mass index
BP: Body pain
CI: Confidence intervals
CRP: C-reactive protein
DAS28: Disease activity score in 28 joints
DMARDs: Disease-modifying antirheumatic drugs
ESR: Erythrocyte sedimentation rate
GH: General health
HADS: Hospital Anxiety and Depression Scale
HAQ-DI: Health Assessment Questionnaire-disability index
IQR: Interquartile range
JSN: Joint space narrowing
MCS: Mental Component Summary
MCPJs: Metacarpophalangeal joints
MH: Mental health
MI: Multiple imputation
OR: Odds ratio
PCS: Physical Component Summary
PIPJs: Proximal interphalangeal joints
PF: Physical function
RA: Rheumatoid arthritis
RE: Role emotional
RF: Rheumatoid factor
RP: Role physical
SD: Standard deviation
SES: Socioeconomic status
SF: Social function
SF-36: The Short Form 36 Health survey
VAS: Visual analog scale
VT: Vitality
