# Erratum to: Factors associated with hand joint destruction in Chinese patients with rheumatoid arthritis

**DOI:** 10.1186/s12891-017-1602-5

**Published:** 2017-05-30

**Authors:** Lijuan Zhang, Jing Wang, Qiuxiang Zhang, Ting Fu, Rulan Yin, Ze Wang, Liren Li, Xianhua Wu, Zhifeng Gu

**Affiliations:** 1 Department of Rheumatology, Affiliated Hospital of Nantong University, 20th Xisi Road, 226001 Nantong, People’s Republic of China; 2 School of Nursing, Nantong University, 19th Qixiu Road, 226001 Nantong, People’s Republic of China; 3 Department of Medical Image, Affiliated Hospital of Nantong University, 20th Xisi Road, 226001 Nantong, People’s Republic of China

## Erratum

After publication of the original article [1] it was brought to our attention that author Jing Wang was incorrectly included as Jing wang. The correct spelling of the name is included in the author list of this erratum and updated in the original article.
